# Effect of Brief Produce Exposure and Unconstrained Grocery Gift Cards on Caregiver Influence on Diet of Elementary Age Children: A Randomized Clinical Trial

**DOI:** 10.1001/jamanetworkopen.2022.12973

**Published:** 2022-05-27

**Authors:** Maninder K. Kahlon, Nazan S. Aksan, Rhonda Aubrey, Jenn Barnes, Nicole Clark, Maria Cowley-Morillo, Lindsey Engelman, Julia Guerra, Alejandro Guevara, Allison Marshall, Deanna M. Hoelscher

**Affiliations:** 1 Department of Population Health, Dell Medical School, The University of Texas at Austin; 2 Boys and Girls Clubs of the Austin Area, Austin, Texas; 3 Michael and Susan Dell Center for Healthy Living, The University of Texas Health Science Center at Houston School of Public Health in Austin

## Abstract

**Importance:**

Children’s diets affect health trajectories but are difficult to influence, especially for resource-constrained families.

**Objective:**

To assess the effectiveness of providing 4 weeks of grocery gift cards and small produce boxes to caregivers on their ability to support healthy shifts in children’s diets.

**Design, Setting, and Participants:**

This 2-group randomized clinical trial was conducted from May to July 2021, with 4 weeks of intervention and follow-up at 8 weeks. Resources were provided curbside at 3 schools, 3 housing sites, and 1 after-school site for use at home. Participants consisted of 1 index child ages 5 to 11 years with 1 index caregiver from 68 low-income families. Data were analyzed from July 2021 through March 2022.

**Interventions:**

During each week for 4 weeks, caregivers were offered 10-lb (4.5 kg) boxes of fruits and vegetables, $10.00 grocery gift cards, an additional $10.00 gift card over the last 3 weeks triggered by a task completion, and a 1-time choice of a $25.00 food preparation tool.

**Main Outcomes and Measures:**

Index child and caregiver diets were measured together over the phone at baseline, 4 weeks, and 8 weeks using the 2019 to 2020 Texas School Physical Activity and Nutrition (SPAN) tool, which measures the number of times food items were eaten over the prior day to report a SPAN Healthy Eating Index (SHEI) score and subscores for specific categories of foods (range, 0-57, with higher scores indicating a more healthful diet).

**Results:**

Among 68 children (mean [SD] age, 8.2 [1.7] years; 35 [51.5%] girls) and caregivers (mean [SD] age, 37.9 [7.9] years; 63 mothers [92.6%]) from primarily low-income families, 26 caregivers were Hispanic or Latino (38.2%), while 18 caregivers were Black (26.4%), 25 caregivers were White (36.7%), and 24 caregivers had more than 1 race (35.3%). Most families were below the federal poverty level (41 of 60 families that reported income [68.3%]). Per participating caregiver, a mean (SD) 2.7 [1.4] fruit and vegetable boxes and $42.35 ($25.46) worth of gift cards were picked up over 4 weeks. Mean (SE) child SPAN SHEI increased from 32.03 (0.62) times/d to 33.75 (0.69) times/d at 4 weeks (ie, postintervention) and 34.03 (0.69) times/d 4 weeks later (ie, at 8 weeks of follow-up). Mean (SE) child fruit and vegetable intake increased from 5.31 (0.47) times/d to 5.78 (0.51) times/d postintervention and 6.03 (0.51) times/d at follow-up. Children in the control group did not have improved diet (overall mean [SE] SHEI: 31.48 [0.58] times/d at baseline, 31.68 [0.54] times/d postintervention, and 31.81 [0.52] times/d at follow-up; mean [SE] fruit and vegetable intake: 5.21 [0.45] times/d at baseline, 4.77 [0.45] times/d postintervention, and 4.68 [0.41] times/d at follow-up). Compared with children in the control group, mean SHEI was increased for children in the intervention group by 2.07 times/d postintervention and 2.23 times/d at follow-up. Improvements as a function of program dose were statistically significant for child SHEI (*P* = .01) and fruit and vegetable intake (*P* = .03). No significant changes in caregiver diets were found.

**Conclusions and Relevance:**

This study found that easily accessed fruits and vegetables and unconstrained grocery store cards provided directly to caregivers over 4 weeks resulted in improvements in child diet, which were sustained over 4 additional weeks. Future work may investigate whether diet improvement from a brief intervention optimized for caregiver flexibility reflects a natural maximum or potential for greater improvements on extension.

**Trial Registration:**

ClinicalTrials.gov Identifier: NCT04827654

## Key Points

**Question:**

Can easy access to produce and flexible resources over 4 weeks among caregivers result in improvements in the diet of their children?

**Findings:**

In this randomized clinical trial of 68 children from primarily low-income families, produce boxes and $10 grocery gift cards provided to caregivers weekly for 4 weeks resulted in a mean increase in net healthy food items eaten per child by 2.07 times/d at 4 weeks and sustained at 2.23 times/d at 8 weeks compared with children in the control group. The results were statistically significant as a function of dosage.

**Meaning:**

These results suggest that brief but flexible support for caregivers to try healthier diets for their children may have a measurable impact on child diet, with evidence for sustained effects beyond the brief program period.

## Teaser

This randomized clinical trial investigates the effect of grocery gift cards and fruit and vegetable boxes provided to caregivers on healthfulness of diets among their children.

## Introduction

To influence diets of children, caregivers who are at low incomes need resources to access and use healthy food, including compensation for waste and time to support experimentation with their children.^1^ Programs that address such barriers have found that, with support, caregivers can foster improvements in their children’s diets. For example, a produce prescription program (Produce Rx) provided vouchers during clinic visits to reduce barriers to access. Over 8 to 12 months, researchers saw an increase in daily fruit and vegetable consumption per child (mean age, 10 years) by 0.26 cups (0.06 L).^2,3^ In another approach, fruits and vegetables were directly delivered in boxes at schools. Over 8 weeks, researchers found an increase in daily fruit and vegetable consumption per child (mean age, 6 years) of 0.24 cups (0.06 L).^4^

Site of delivery and format of resource matter. Prescriptions at a clinic explicitly and implicitly reinforce health benefits. However, families could be inconvenienced by having to visit a clinic to collect resources.^5,6,7^ At schools, programs can be better integrated into existing routines and involve greater family participation, with potential for broad benefits.^8^ Additionally, providing produce directly reduces barriers to access and incentivizes families to try unfamiliar produce. However, providing a voucher or gift card allows families to choose produce better aligned with cultural preferences and children’s tastes^5,6^ and provides flexibility in making trade-offs.^9^ For example, caregivers may use vouchers to pay slightly higher mean prices while consolidating shopping at a single store to gain time with their children.

We wanted to assess how much and how rapidly we could influence children’s diets by simultaneously removing as many barriers as possible for a caregiver to implement their own strategies. We began by integrating the intervention into caregiver routine as they drove up for an existing curbside program delivered by the Boys and Girls Clubs of the Austin Area (BGCAA) in Texas. We provided fruit and vegetable boxes directly and gift cards for a commonly used grocery store. We facilitated preparation of healthy food by providing a kitchen tool, recipes, and cooking tips, reinforcing healthy eating goals without prescribing specific activities. Hence, we reduced barriers to access, provided instrumental support, offered options to allow for individual strategies, and consistently reinforced healthy eating goals. Our objective was to assess the extent of improvement in child diet that could be achieved by 4 weeks and sustained by 8 weeks with this program.

## Methods

This study is a randomized clinical trial of a pilot program delivered to caregivers for 4 weeks from May 4 to 27, 2021 (trial protocol in Supplement 1). The study was approved by the Institutional Review Board at the University of Texas at Austin and follows the Consolidated Standards of Reporting Trials (CONSORT) reporting guideline. Verbal consent was obtained from all caregivers and children.

### Program Setting

BGCAA operates after-school programming in schools in low-income neighborhoods, in public housing, and through a central home club. Because after-school programming was curtailed owing to the COVID-19 pandemic, BGCAA had begun providing bags containing children’s activities and snacks (eg, beef sticks, granola bars, and dried cranberries) through a weekly curbside distribution called Club on the Go (COTG) at BGCAA’s 22 sites. Our program was implemented via COTG at 7 sites, including 3 school, 3 housing, and 1 home club sites that other pandemic programs were not covering.

### Program Protocol

BGCAA staff provided program elements to caregivers weekly for 4 weeks as caregivers drove up to receive COTG bags. The program framework is described in Table 1. We reduced barriers to resources by providing a small box of fruits and vegetables weekly (6-8 items determined by vendor, always including fruit but emphasizing vegetables, weighing <10 lbs [4.5 kg]), a $10.00 nonexpiring grocery gift card (gift card), and an additional $10.00 weekly for 3 weeks if caregivers completed a small task (described in a subsequent section), for a total receivable cash value of $70.00 and produce of less than 40lbs (18.1 kg). We provided instrumental support weekly via an envelope in the fruit and vegetable box that included 3 bilingual, culturally relevant recipes with easy-to-source ingredients customized to the box’s contents and 1 cooking tip sheet (eg, on how to store produce). In the first week, participants also received a food preparation tool (worth <$25.00) that they had selected at baseline; options were a knife set, blender, spice kit, set of mixing bowls, and children’s chef kit (including chef hat, apron, and vegetable cutters in engaging shapes). We supported caregiver individual strategies by providing a choice of tool and produce and a gift card for a popular grocery store chain that sold household, pharmacy, and food items. We reinforced program goals in multiple ways, including via our partner, BGCAA, already recognized by our participants as promoting healthy lifestyles, and in materials and resources provided. Additionally, in weeks 1 to 3, fruit and vegetable boxes contained a brief survey asking about produce the caregiver and child had tried the prior week (ie, a goals survey). If caregivers returned the survey the following week, they received an additional $10.00 gift card (in weeks 2-4). After the program ended, during the following 4 weeks (ie, follow-up), caregivers in the intervention group were mailed two $5 gift cards as small reinforcing incentives.

**Table 1.** Program Framework and Elements

Approach	Program element
**Caregiver anchor**	
Reduce barriers	Integrate into caregiver routine One <10-lb fruit and vegetable box with 6-8 items (weekly, 4 wk) $10 Gift cards (weekly, 4 wk) plus $10 if task completed (weekly, 3 wk)
Provide instrumental support	1 Kitchen tool,<$25 value (first week) 3 Recipes customized to contents of produce box (weekly for 4 wk) 1 Cooking tip sheet (weekly for 4 wk)
Support individual strategies	Choice of kitchen tool from 5 options (first week) Produce box and grocery gift cards provided (weekly for 4 wk) Unconstrained grocery gift card
**Program anchor**	
Reinforce healthy eating goal	Trusted implementation partner already associated with health goals Multiple reinforcements of program goal Specific incentive to complete small task (survey) that reminds caregiver of goal

### Recruitment, Consent, and Randomization

Caregivers, primarily BGCAA members, learned about the program from BGCAA staff during child pickup or through flyers in COTG bags, word of mouth, or proactive outreach by phone. Inclusion criteria included having at least 1 child in grades kindergarten through 5, being able to come to the pickup site of choice for 4 weeks, and being able to read and write in English or Spanish. Exclusion criteria included any medical condition that would make it difficult to make dietary changes, as assessed by caregiver self-report. Verbal consent was obtained on the phone from the index caregiver first and then from the index child. Study data were collected and managed using REDCap electronic data capture tools (REDCap).^10^ The biostatistician (N.S.A.) used the ralloc package of Stata statistical software to generate a randomization scheme in a 1:1 ratio between control and intervention groups in blocks of 2 and 4 with stratification by sex. This scheme was uploaded into REDCap and run after each participant’s baseline data collection for group assignment in queue. After randomization, individuals in the intervention group were notified with program details, while those in the control group were notified that they would receive an $80.00 gift card mailed to them after their final follow-up data collection in 8 weeks. Participants in the control group received no other resources until then.

### Measurement

Study measurements were taken in English or Spanish on the phone through self-report from the index caregiver and separately from the index child with the caregiver helping as necessary. Four research associates (including M.C.M., A.G., and J.G.) collected data at baseline (ie, before the first pickup on May 4, or March 31 through April 30), postintervention (ie, beginning after the final pickup on May 25 and continuing during the following 2 weeks, or May 26 through June 9), and follow-up (ie, beginning week 8 and continuing through the end of week 9, or June 24 through July 1). Research associates were blinded to the allocation group, except for the final questions in follow-up assessing program satisfaction (in the intervention group only).

### Measures

At baseline, we collected information on demographics, food insecurity, and participation in the Supplemental Nutrition Assistance Program (SNAP) and Special Supplemental Nutrition Program for Women, Infants, and Children (WIC).^11^ Race and ethnicity data were collected via self-report to support future scaling of the program. The options provided were American Indian or Alaska Native, Asian, Black or African American, Native Hawaiian or other Pacific Islander, White, more than 1 race, or unknown or not reported for race and Hispanic or Latino, not Hispanic or Latino, and unknown for ethnicity. At baseline, postintervention, and follow-up, we collected dietary intake information and self-assessments of health behaviors and preferences. Program satisfaction was assessed for individuals in the intervention group at follow-up.

#### Diet

Dietary intake was assessed using food frequency items from the 2019 to 2020 Texas School Physical Activity and Nutrition (SPAN) study instrument, which has been previously tested for reproducibility and validity for families with similar income and demographics and children aged 8 to 12 years.^12,13,14,15,16^ SPAN measures dietary behaviors in reference to consumption the day prior by asking, “Yesterday, did you eat [item]?”, with response options of 0, 1, 2, or 3 times, phrased as, “No, I didn’t eat any [item] yesterday,” (0 times) and, “Yes, I ate [item] 1 time yesterday,” (1 time), and so forth.

SPAN includes 2 fruit items (whole fruit and 100% fruit juice) and 5 vegetable items (starchy vegetables, orange vegetables, green vegetables, other vegetables, and beans and legumes). We also measured consumption of healthful items (plain milk and yogurt or yogurt drinks) and 10 unhealthful items (french fries, chips, and crackers; snack bars; frozen desserts; baked sweets; candy; flavored milk; and punch, flavored drinks, sports drinks, regular sodas, and energy drinks). For caregiver diet, fruit and vegetable items and unhealthful items (snack bars, frozen desserts, baked sweets, and candy) were assessed.

We generated 3 scores for children and caregivers on frequency of intake as reported for the previous 24 hours. First, a score for overall diet or SPAN Healthy Eating Index (SHEI), which gives the number of times food items were eaten over the prior day, was calculated. Each item was scored 0, 1, 2, or 3 (for 3 or more) times items eaten per day and summed. Thus, scores ranged from 0 to 57 times that items were eaten per day for children for 19 items, including 7 fruit and vegetable items, 2 milk and yogurt items, and 10 reverse-coded unhealthy items.^17^ We also calculated subscales for fruits and vegetables (7 items, with scores from 0-21 times items were eaten per day) and unhealthy items (10 items for children, with scores from 0-30 times items were eaten per day).

The normalized SHEI has demonstrated construct validity.^17^ We report raw SHEI scores so changes can be more easily interpreted, with a difference of 1 interpreted as a 1 time/d difference in the net of healthy and unhealthy foods eaten. Similarly, for fruit and vegetable and unhealthy subscales, scores represent the number of times items in a particular category were consumed in the prior day. For comparison, 1 time/d is considered roughly equivalent to 1 serving or one-half cup (0.1 L).

#### Dose

We constructed a dose variable that counted the number of elements a caregiver engaged with every week over 4 weeks of the program, for a total of 11 points. These included picking up a fruit and vegetable box (maximum, 4 points) or the envelope with gift card (maximum, 4 points) and returning goal surveys to receive the extra gift card (maximum, 3 points). For 2 families that dropped out before the end of the 4-week program, the denominator was adjusted for number of possible elements they could fulfill.

### Statistical Analysis

We used linear mixed-effect regressions to test changes in child SPAN scores obtained over 3 assessment waves in 2 groups. Research questions concerned the interaction of time with the grouping indicator on the following SPAN scales separately for caregiver and child: SHEI, fruit and vegetable intake, and overall unhealthy food intake. To control for inflations of type I error rate, we planned to test the omnibus interaction effect based on the overall SPAN index and, if significant, test follow-up interactions on 2 subscales: fruit and vegetable and unhealthy food intake. We targeted a sample size of 120 individuals with 80% power to detect an effect size of *f* = 0.14 for this omnibus interaction effect, testing differential trajectories for the control and intervention groups, with an α = .05 and assuming a conservative level of rank-order stability among successive assessment waves, with *r* = 0.30. All statistical tests were 2-sided. Analyses were conducted using Stata statistical software version 16.1 (StataCorp). Data were analyzed from July 2021 through March 2022.

We recruited until the end of the school year but did not achieve the targeted sample size. Therefore, we added a second mixed linear analysis replacing the grouping indicator with a dose variable that quantified uptake of intervention elements over the 4-week program. The dose variable was set to 0 for participants in the control group and varied from 0 to 1 for participants in the intervention group, reflecting the proportion of available program elements the caregiver took up. Assessing the effect of interest using the dose variable provides greater sensitivity and precision than the grouping variable and hence more power. For the dose variable among 68 participants in the control group, the mean (SD) dose was 0.34 (0.42) of program elements; for 35 intervention participants, the mean (SD) does was 0.65 (0.36) of program elements. Time was modeled as elapsed days between baseline (set to 0 days for all participants) and subsequent assessments. In addition, mixed linear regressions modeled 7 sites as a clustering variable.

## Results

**Figure 1.**
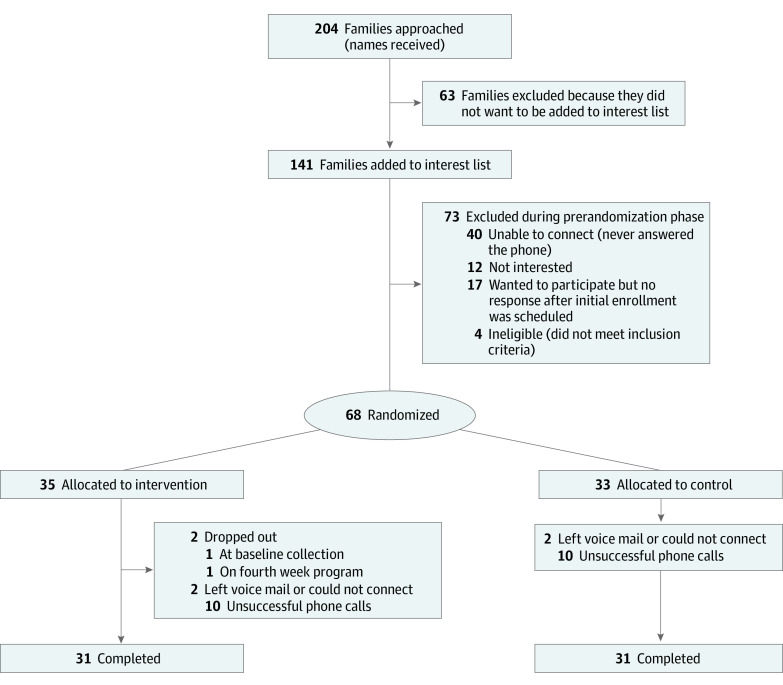
Study Flowchart

The CONSORT diagram (Figure 1) shows that 204 families were approached and 68 families participated in the study. In the intervention group, 31 of 35 families (88.6%) completed the program. Children’s ages ranged from 5 to 11 years, and the population included girls and boys (Table 2). Among 68 children (mean [SD] age, 8.2 [1.7] years; 35 [51.5%] girls) and caregivers (mean [SD] age, 37.9 [7.9] years; 63 mothers [92.6%]), 26 caregivers were Hispanic or Latino (38.2%), while 1 caregiver was Asian (1.4%), 18 caregivers were Black (26.4%), 25 caregivers were White (36.7%), and 24 caregivers had more than 1 race (35.3%). Most caregivers (41 of 60 individuals reporting income [68.3%]) reported earning less than $36 000 per year. Less than half of caregivers (30 individuals [44.1%]) reported being on SNAP, and most caregivers (41 individuals [60.3%]) reported worrying about food running out in the prior 12 months. Most families (41 families that reported income) were below the federal poverty level given the ratio of income to household size. Households usually included more than 1 child, with 30 families (44.1%) including more than 2 children.

**Table 2.** Baseline Demographic Characteristics

Characteristic	Participants, No. (%) (N = 68)		
	Intervention (n = 35)	Control (n = 33)	Total
**Child characteristic**			
Age, mean (SD), y	8.8 (1.7)	7.64 (1.5)	8.23 (1.7)
Sex			
Girls	19 (54.2)	16 (48.5)	35 (51.5)
Boys	16 (45.7)	17 (51.5)	33 (48.5)
**Caregiver characteristic**			
Age, mean (SD), y	37.6 (8.4)	38.3 (7.6)	37.9 (7.9)
Mothers	32 ( 91.4)	31 (94.1)	63 (92.6)
Hispanic	14 (40.0)	12 (36.4)	26 (38.2)
Race			
Asian	1 (2.9)	0	1 (1.4)
Black or African American	11 (31.4)	7 (21.2)	18 (26.4)
White	13 (37.1)	12 (36.4)	25 (36.7)
≥1 Race	10 (28.5)	14 (42.4	24 (35.3)
**Family characteristic**			
Monthly income, $ (n = 60)			
>6500	5 (16.7)	2 (6.7)	7 (11.7)
6500-5000	1 (3.3)	3 (10.0)	4 (6.7)
4999-3000	3 (10.0)	5 (16.7)	8 (13.3)
2999-1700	10 (33.3)	12 (10.0)	22 (36.7)
1700-800	10 (33.3)	8 (26.7)	18 (30.0)
<800	1 (3.3)	0	1 (1.7)
Participation in food program			
Currently enrolled in SNAP	15 (42.9)	15 ( 45.5)	30 (44.1)
Currently or ever enrolled in WIC	20 (57.1)	19 ( 57.6)	39 (57.4)
Food scarcity past 12 mo			
Worry about food running out	23 (65.7)	18 (54.5)	41 (60.3)
Food actually ran out	19 (54.2)	16 (48.5)	35 (51.5)
Time child lives with caregiver, %			
75	2 (5.7)	0	2 (2.9)
90	1 (2.9)	0	1 (1.4)
100	32 (91.4)	33 (100)	65 (95.6)
No. of children living with caregiver			
≤2	22 (62.9)	16 (48.5)	38 (55.9)
3-4	11 (31.4)	13 (39.4)	24 (35.3)
>4	2 (5.7)	4 (12.1)	6 (8.8)
No. of adults in household			
1	9 (25.7)	8 (24.2)	17 (25.0)
2	25 (71.4)	16 (48.5)	41 (60.3)
≥3	1 (2.9)	9 (27.3)	10 (14.7)

Abbreviations: SNAP, Supplemental Nutrition Assistance Program; WIC, Special Supplemental Nutrition Program for Women, Infants, and Children.

**Figure 2.**
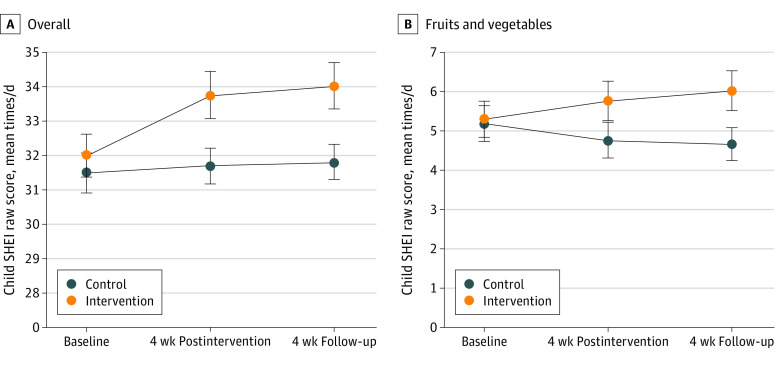
Child Texas School Physical Activity and Nutrition (SPAN) Healthy Eating Index (SHEI) Score Over Time Means and SEs are presented.

Table 3 describes results. Mean (SE) child SHEI score, which summarizes net healthfulness of diet, increased in the intervention group from 32.03 (0.62) times/d at baseline to 33.75 (0.69) times/d postintervention and 34.03 (0.69) times/d at follow-up (time × group *P* = .08; time × dose *P* = .01). This was an increase by 1.72 times/d postintervention and 2.00 times/d at follow-up. Mean (SE) SHEI for children in the control group did not increase significantly: 31.48 (0.58) times/d at baseline, 31.68 (0.54) times/d postintervention, and 31.81 (0.52) times/d at follow-up (Figure 2). Panel A of Figure 2 depicts these changes of SHEI over time. The Pearson correlation between dose variable and raw difference score for improvements from baseline to follow-up was *r*(62) = 0.3059 (*P* = .02). Pairwise differences between groups at each assessment wave are also shown in Table 3. Within-group change in SHEI from baseline to 8-week follow-up with listwise deletion had a mean (SE) of 1.90 (0.749) times/d (95% CI, 0.37 to 3.43 times/d) in the intervention group and 0.322 (0.609) times/d (95% CI, −0.92 to 1.57 times/d) in the control group. The difference between these within-group differences (SE) was 1.58 (0.97) times/d (95% CI, −0.35 to 3.51 times/d). Mean (SE) fruit and vegetable intake increased from 5.31 (0.47) times/d at baseline to 5.78 (0.51) times/d postintervention and 6.03 (0.51) times/d at follow-up (time × group *P* = .08; time × dose *P* = .03). This was an increase by 0.47 and 0.72 times/d, respectively. In the control group, mean (SE) intake did not increase significantly: 5.21 (0.45) times/d at baseline, 4.77 (0.45) times/d postintervention, and 4.68 (0.41) times/d at follow-up (Table 3). Figure 2B depicts these changes over time. No trends were statistically significant for unhealthy food items. Unlike the results for children, there were no statistically significant trends for caregiver diet.

**Table 3.** Food Intake Outcomes and Interactions Terms

Group	Food intake, mean (SE), times/d			Interaction effects^a^					
				Days × group			Days × dose		
	Baseline	4 wk	8 wk	*P* value	ICC, site (95% CI)	ICC, person × site (95% CI)	*P* value	ICC, site (95% CI)	ICC, person × site (95% CI)
**Child**									
Raw SHEI									
Intervention	32.03 (0.62)	33.75 (0.69)	34.03 (0.69)	.08	0.16 (0.04 to 0.47)	0.36 (0.19 to 0.57)	.01	0.19 (0.05 to 0.51)	0.4 (0.22 to 0.61)
Control	31.48 (0.58)	31.68 (0.54)	31.81 (0.52)						
Difference, mean (95% CI)	0.54 (−1.15 to 2.24)	2.07 (0.32 to 3.83)	2.23 (0.51 to 3.95)	NA	NA	NA	NA	NA	NA
Fruits and vegetables									
Intervention	5.31 (0.47)	5.78 (0.51)	6.03 (0.51)	.08	0.06 (0.01 to 0.32)	0.42 (0.27 to 0.58)	.03	0.07 (0.01 to 0.34)	0.43 (0.29 to 0.59)
Control	5.21 (0.45)	4.77 (0.45)	4.68 (0.41)						
Difference, mean (95% CI)	0.10 (−1.20 to 1.40)	1.01 (−.36 to 2.38)	1.35 (0.05 to 2.66)	NA	NA	NA	NA	NA	NA
Unhealthy foods									
Intervention	4.74 (0.40)	3.53 (0.44)	3.52 (0.42)	.51	0	0.22 (0.10 to 0.42)	.23	0	0.23 (0.10 to 0.42)
Control	5.03 (0.41)	4.42 (0.46)	4.26 (0.40)						
Difference, mean (95% CI)	−0.29 (−1.43 to 0.86)	−0.89 (−2.15 to .38)	−0.74 (−1.89 to 0.42)	NA	NA	NA	NA	NA	NA
**Caregiver**									
Raw SHEI									
Intervention	15.83 (0.47)	16.59 (0.52)	16.90 (0.40)	.73	0.07 (0.01 to 0.29)	0.32 (0.18 to 0.51)	.10	0.08 (0.02 to 0.31)	0.34 (0.20 to 0.52)
Control	15.39 (0.43)	15.35 (0.44)	16.03 (0.43)						
Difference, mean (95% CI)	0.43 (−0.84 to 1.71)	1.24 (−0.13 to 2.61)	0.87 (−0.30 to 2.04)	NA	NA	NA	NA	NA	NA
Fruits and vegetables									
Intervention	5.23 (0.47)	5.72 (0.53)	5.90 (0.43)	.85	0.1 (0.02 to 0.34)	0.43 (0.28 to 0.59)	.15	0.1 (0.02 to 0.36)	0.44 (0.29 to 0.60)
Control	15.83 (0.47)	16.59 (0.52)	16.90 (0.40)						
Difference, mean (95% CI)	0.44 (−0.88 to 1.76)	1.27 (−0.12 to 2.65)	0.71 (−0.60 to 2.01)	NA	NA	NA	NA	NA	NA

Abbreviations: ICC, intraclass correlation; NA, not applicable; SHEI, Texas School Physical Activity and Nutrition (SPAN) Healthy Eating Index.

^a^ *P* values are for interaction effects from 2 linear mixed effect regressions, days × group or days × dose, and associated ICCs.

Intervention elements (ie, dose) had a large range; among 34 participants who began the program, 2 participants never picked up anything and 5 participants picked up the maximum incentives (ie, 4 fruit and vegetable boxes and 7 gift cards worth $10.00 each). Goal surveys were returned the following week 47 of 69 times surveys were given to participants (68.1%). A mean (SD) of 2.7 (1.4) fruit and vegetable boxes and $42.35 ($25.46) worth of gift cards were picked up per participant. Intervention uptake did not differ by SNAP recipient status but did differ by WIC recipient status. Participants who were never WIC recipients used intervention elements to a greater extent than those who were WIC recipients (*r*[35] = −0.34; *P* = .047). There was a significant trend for families at lower federal poverty levels to uptake more elements of the intervention (*r*[30] = 0.372; 95% CI, 0.014-0.646; *P* = .04). Among 31 participants who completed the study in the intervention group and answered an open-ended question about what items gift cards were used to purchase, 29 participants (93.5%) described using gift cards to purchase food but 2 participants (6.5%) mentioned nonfood household necessities.

Of 5 food preparation incentives, among 35 participants who answered the question, 16 participants (45.7%) in the intervention group preferred the children’s cooking kit (26 of 67 participants total who answered the question [38.8%]) and 10 participants (28.6%) in the intervention group preferred the blender (20 participants total [29.9%]) over the mixing bowls, knife set, and spice kit. At baseline, among 68 participants who answered the question, 44 participants (64.7%) preferred to receive a grocery card and 24 participants (35.3%) preferred a fruit and vegetable box. We reassessed for the intervention group at the end of the program and detected no evidence of these preferences changing (χ^2^_1_ = 1.29; exact *P* = .45, McNemar).

## Discussion

This randomized clinical trial found that a 4-week program that provided multiple resources and instrumental support to caregivers within their regular routine improved the diet of elementary-aged children, sustaining over the following 4 weeks. At baseline, children (mean age, 8 years) consumed fruits and vegetables from 5.21 to 5.31 times/d, or 5.21 to 5.31 servings/d. A serving is operationalized as 0.5 cups (0.1 L); thus at approximately 2.5 cups (0.6 L), this was below the recommended 4 cups (0.9 L) for an 1800-calorie diet.^18^ Over 4 weeks of the program and 4 weeks of follow-up, children’s overall diet (SHEI) showed improvements in intake of net healthy foods, from 32.03 times/d to 33.75 times/d postintervention and 34.03 times/d at follow-up, or by 1.72 and 2.00 times/d, respectively (0.86-0.99 cups/d [0.20-0.23 L/d]). This increase reflected a statistically significant increase in fruit and vegetable intake, from 5.31 times/d to 5.78 times/d postintervention and 6.03 times/d at follow-up, or by 0.47 and 0.72 times/d, respectively (0.23-0.36 cups/d [0.05-0.09 L/d]), and no statistically significant change in unhealthy food intake.

Programs that target caregivers to improve child diet are heterogenous but generally focus on providing material resources or education, with a combination being most effective.^2,3,4,19,20,21,22^ We benchmarked our results against 2 large studies reporting the greatest impacts; both studies anchored on providing resources.^2,4^ In 1 study^4^ with a comparison group, 407 caregivers at 8 schools with children aged a mean of 6 years participated in a cooperative program at school sites helping to pack and then take home 30-lb (14 kg) fruit and vegetable boxes weekly, with results measured after 8 and 16 weeks over 2 school semesters. In another study,^2^ without a comparison group, 883 caregivers of children with overweight and obesity received education and monthly produce prescriptions at clinics over a mean of 3 months and a mean of $361 redeemed at farmers markets. These programs resulted in similar scale improvements in children’s fruit and vegetable intake of approximately 0.25 cups/d (0.06 L/d), similar to the results in our study. However, our program was brief and easy to implement, benefiting, we believe, from multiple pathways for engagement, including exposure to unfamiliar items and use of existing preferences.

Effects sustained over an additional 4 weeks after the program ended. During the follow-up period, although fruit and vegetable boxes were no longer available, gift cards could still be used. However, there was no requirement to purchase healthy food. Caregivers also received 2 additional $5.00 gift cards in the mail as small incentives, but no other reinforcement of program goals or additional instrumental support was provided. In fact, the BGCAA curbside COTG program ended at the same time as our program, with the beginning of summer. Although we cannot make conclusions about why the results sustained, it is likely that families were still using the gift cards. In addition, we hypothesize that allowing caregivers flexibility to implement their own strategies may also support longer-term change. For example, we found at baseline that 54% of caregivers preferred receiving gift cards while 38% preferred fruit and vegetable boxes, and these preferences did not change at 4 weeks after caregivers had tried both options. In a program that offered 1 of these options, it is possible we would see reduced impact. In addition, gift cards did not limit purchases to healthy foods. Thus, symbolically and practically, the program respected caregiver agency, and findings suggest that healthy items may not need to be prescribed to achieve healthier outcomes. It was also telling that when offered a choice of food preparation tool, 39% of caregivers chose the children’s chef kit, which was less practical but that aligned with goals for improving their child’s diet. Future work may unpack the differential impact of agency in sustaining improvements in diet as suggested by our findings.

Given that this program achieved similar improvements in diet in a shorter period than other programs, it is possible that effects could be enhanced through a longer intervention. On the other hand, we may have reached a natural maximum for individually targeted programs. We know that structural improvements, such as improvements to school lunches, food retail environments, standards of pay to support living wages and childcare, and parental and sick leave, play a pivotal role in improving children’s diets.^1,23^ Perhaps brief individually targeted programs optimized for caregiver agency combined with the launch of food-related policy or other structural changes may accelerate adoption by caregivers and their children for multiplicative impact.

### Limitations

Our study has several limitations, especially small sample size. In addition, we recruited one-third of the families to whom the program was offered, suggesting that the program was not as convenient as we had hoped. Curbside pickup limited the program to families with transportation. Food frequency questionnaires have self-report bias even if ascertainment bias was minimized through masking. We were unable to detect caregiver diet changes, which would be useful to explore in subsequent work. Additionally, our study was implemented during the COVID-19 pandemic, which may have increased the need for and uptake of the intervention.

## Conclusions

This study found that a brief, 4-week program that gave caregivers agency in making choices between easily accessed resources resulted in improvements in children’s overall diet and fruit and vegetable intake. In adults, a similar scale of improvement through voucher programs has been modeled to show societal, lifetime cost-effectiveness.^24^ Our findings suggest that setting children on healthier trajectories through flexible support for their caregivers may promise significant benefits for families and society.

## Notes

Supplement 1.
Trial Protocol

Supplement 2.
Data Sharing Statement
