# Data Errors in Table 3

**DOI:** 10.1001/jamanetworkopen.2022.31672

**Published:** 2022-08-23

The Original Investigation, “Effect of Brief Produce Exposure and Unconstrained Grocery Gift Cards on Caregiver Influence on Diet of Elementary Age Children: A Randomized Clinical Trial,”^1^ published on May 27, 2022, in *JAMA Network Open*, had errors in 1 row of data in Table 3. The mean (SE) intake of fruits and vegetables for caregivers in the control group should have been 4.79 (0.46) times/d at baseline, 4.45 (0.45) times/d at 4 weeks, and 5.19 (0.49) times/d at 8 weeks. These numbers have been corrected.^1^
