# Supplementary material for: Effect of Brief Produce Exposure and Unconstrained Grocery Gift Cards on Caregiver Influence on Diet of Elementary Age Children: A Randomized Clinical Trial
Source: JAMA Netw Open. 2022 May 27;5(5):e2212973. doi: 10.1001/jamanetworkopen.2022.12973 (PMC9142863; doi:10.1001/jamanetworkopen.2022.12973)
Supplement: Supplement 1. — Trial Protocol [file jamanetwopen-e2212973-s001.pdf]

FORM: IRB Proposal - Standard Submission	
NUMBER	VERSION DATE
HRP-UT901	8/3/2020

GENERAL STUDY INFORMATION

Use for greater than minimal risk studies and minimal risk studies that fit into one or more expedited categories (see Section 5.3 of our Policies & Procedures for details regarding expedited research).

Do NOT submit this form if the study will qualify for exempt review, instead submit HRP-UT902 IRB Proposal – Exempt Submission Form found in the document Library.

If you are only using secondary data that will not be initially collected solely for this research project, use HRP-UT903 Template IRB Proposal Secondary Use form instead.

For studies following a multi-center or sponsor protocol, please use this guidance to assist in your completion of this form.

For questions regarding definitions, policies, or terms referenced below see the policies and procedures manual.

Please note, Word online does not support Word checkboxes. Please download the file and use your desktop version of Microsoft Word.

1 Review Type (Choose one)

*Click on the check box (or double click and type an "X" if using Google Docs) the **one** review type that applies.*

Please note: Expedited Review does not refer to the timeliness of the review of your protocol, but specific categories of research defined by ORHP. If you would like help determining which type of review is most appropriate for your study please contact the Office of Research Support and Compliance: https://research.utexas.edu/ors/about-ors/contact-us/.

a ☐ **Full Board Review – Greater than Minimal Risk Research**

b ☒ **Expedited Review – Minimal Risk Research**

2 Research Hypothesis

To input text, click in the light grey area below.

We are interested in maximizing the consumption of fruits and vegetables (F&V) in elementary school children by increasing the access to F&V through providing them in the home environment via direct delivery (F&V bag & recipes) and a gift card to purchase preferred choices. This also decreases barriers to trying F&V and switching to a more a F&V-focused diet, e.g., access, cost, knowledge of what to do with fruits and vegetables by their families in household diet, and a period of time to try and get used to more F&V in diet without worrying about access and cost.

We hypothesize that at the end of 4 weeks, children in families that were given access to a F&V produce box with associated recipes, a gift card to purchase preferred choices, and a cooking incentive kit, consumed significantly better diets as measured with a diet composite score compared with children in families who did not receive increased F&V access.

21

3 Study Background

To input text, click in the light grey area below.

Financial incentives have been shown to increase F&V adoption by adults and in other cases by children in families that received the incentive. When applied as a large scale rebate program of 30% cash back for targeted F&V purchases, consumption increased by an average of 0.24 cups per day for each individual on SNAP for whom this was tested (Olsho et al, 2016). In other work, provision of F&V together with recipes and guidelines to cook with them were used to incentivize their utilization in diets for families with children in elementary schools (Sharma et al, 2016)). Here, for a 16 week program a 0.48 cups per day increase was seen, with a significant increase at 8 weeks of the program that was then sustained for an additional 8 weeks (for a total of 16 weeks of the program conducted over two semesters).

Other models for F&V delivery that have been tested include boxes from food pantries (Seligman et al, 2018) or F&V vouchers provided as "prescriptions" (just like medical prescriptions) during clinic visits (Ridberg et al, 2019). However both models require effort to integrate into the site and schedule of a food pantry or a clinic. The examples of F&V delivery shared above, either a rebate on a SNAP card that is already being used or F&V delivery to schools where caregivers are already present, are both attractive because they reduce barriers to adoption by integrating into the regular cadence of lives. To maximize effectiveness, the program we have designed ties to current activities of families and allows them to try both a box and a gift card. The approach draws from an equity and social ecological framework as described by Kumanyika (2019). Our long-term goal is to provide options that may be otherwise harder to access for lower-resourced families that help them increase their consumption of F&V. Our eventual goal is to improve clinical measures of health outcomes, but we focus here first on maximizing consumption of F&V first.

To maximize the likelihood of F&V pickup and use in daily diets, we wish to test the inclusion of F&V into diet after a 4-week convenient provision of F&V. Because of COVID-19, our partner agency Boys and Girls Club, with whom we already have a strong relationship, designed a program called "Club on the Go" where families could drive up and pick up a range of educational and nutritional components, from flyers, activities for children to snacks. They plan to continue this program throughout the school semester next year. This provides an opportunity to test how easy, customized household access to F&V – by providing pre-made F&V bag and a gift card to parents who have already chosen to drive up to pick up bags from "Club on the Go" – might maximize intake into diet over 4 weeks.

The 4-week intervention is shorter than most, but we will focus on maximizing adoption through integrated recipes, the use of existing relationships between BGC site staff and the families, and a phone number that already exists to allow for questions as they arise. We will also try to maximize the impact of this intervention during the follow-up period, by providing small but meaningful continued incentives to eat F&V even after we stop delivering F&V and gift cards.

Finally, this work will inform a study, approved (IRB# 2020-05-0001) that we had to postpone due to COVID-19 and is now slated to begin in Fall 2021. What we learn here will allow us to incorporate this F&V strategy as part of a multilevel program with other components to maximize health outcomes in girls 8-11years at these school sites.

References

- Kumanyika S K. A Framework for increasing equity impact in obesity prevention. Am J Public Health. 2019;109(10):1350-1357. doi:10.2105/AJPH.2019.305221.
- Olsho L EW, Klerman J A, Wilde P E, Bartlett S. Financial incentives increase fruit and vegetable intake

among Supplemental Nutrition Assistance Program participants: a randomized controlled trial of the USDA healthy incentives pilot. Am J Clin Nutr. 2016;104(2):423-35. doi:10.3945/ajcn.115.129320.

- Ridberg R A, Bell J F, Merritt K E, Harris D M, Young H M, Tancredi D J. Effect of a fruit and vegetable prescription program on children's fruit and vegetable consumption. Prev Chronic Dis. 2019;16(73):180555. doi:10.5888/pcd16.180555.
- Seligman HK, Smith M, Rosenmoss S, Marshall MB, Waxman, E. Comprehensive Diabetes Self-Management Support From Food Banks: A Randomized Controlled Trial American Journal of Public Health 2018 108, 1227_1234, doi:10.2105/AJPH.2018.304528
- Sharma S V, Markham C, Chow J, Ranjit N, Pomeroy M, Raber M. Evaluating a school-based fruit and vegetable co-op in low-income children: a quasi-experimental study. Prev Med. 2016;91:8-17. doi: 10.1016/j.ypmed.2016.07.022.

22

4 Design and Methodology

Provide information regarding study design or data collection methodologies. Details regarding protocol specific research procedures will be discussed in a later section.

To input text, click in the light grey area below.

Design: Randomized Controlled Trial

Randomization: Participants (each household of a reference child) will be randomized in a 1:1 ratio between control and intervention in blocks of 2 and 4.

Intervention: Families in the intervention arm will receive a program of 4 weeks of access to F&V and a gift card, provided weekly through the Boys and Girls Club "Club on the Go" sites. Families in the intervention arm will also get an extra Gift card if they (on weeks 2, 3 and 4)they provide responses to a card that allows them to track their own progress on goals for healthier eating.

Control: The control arm will receive no additional F&V access or gift card until the brief follow-up period at 8 weeks, at which point they will receive an equivalent of the 4 weeks of F&V received by the intervention arm as a Gift card.

Primary Outcome: Diet of a reference child, grades K to 5 who's family member picks up weekly "Club on the Go" bags distributed by Boys and Girls club at specific sites. Measured by a previously validated tool to measure diet recall by elementary-aged children used by one of the investigators (Hoelscher) (SPAN 2nd Grade, see below).

Secondary outcomes:

1. Caregiver diet (SPAN 8th Grade, see below)
2. Process measure (only intervention): Choice made between a selection of additional cooking incentives offered at the start of the program (potential incentives include: kitchen equipment, cookbook, spice kit)

In addition, we are interested in family's perceptions of what was useful in the program to support their desire for their families to eat healthier.

Measures:

- Child & Caregiver Diet: We will use a subset of questions from the Texas School Physical Activity and Nutrition (Texas SPAN for 2nd Grade for children; for 8th grade for caregiver) survey tool, that has been validated for use with parents and children by one of the investigators on the team (Thiagaraja et al, 2006, Penkilo, George & Hoelscher, 2008, Hoelscher et al, 2003) ("SPAN").
- All SPAN measurements will be collected with caregiver and child together, over the phone.
- Measures will be compared at timepoints, baseline (-4 weeks), post (4 weeks) and follow-up (8 weeks), between Control (no F&V) and Intervention (4 weeks of F&V access choice)

**Outcome Measures**	**Collection Tool**	**Frequency**
Demographics	Redcap Survey	Baseline
Reference child diet	SPAN 2nd Grade [SPAN 17-100]	Baseline, 4 weeks, 8 weeks
Caregiver diet	SPAN 8th Grade [SPAN 8-47]	Baseline, 4 weeks, 8 weeks
Fruits & Vegetable Access Method Preference	Redcap Survey	Baseline, 4 weeks.
Program Improvement and Satisfaction	Redcap Survey	8 weeks
Social Needs Screening	AAFP Social Needs Assessment	Baseline
Health Goals	Redcap Survey	Intervention weeks 2, 3 and 4

Randomization and Blinding

Randomization: participants will be randomized on a 1:1 (control:intervention) ratio after their baseline assessments have been taken. Both arms will receive the same value of F&V access, but the control arm will receive a gift card at the end of the study period.
Blinding: The research team taking post and follow-up measurements will be blind to the arm that the interviewee is in. At the end of the follow-up (8 week) questions, the intervention arm will receive a few additional questions more generally about their assessment of the program.

Measurement Approach

Consistency in who measures: Because of the timeframe constraints for taking measurements, we expect to include 4 research associates to take measurements. Whenever possible, each research associate will remain with the same families to keep consistency at all 3 measurement time points.

Measurements to be taken by research team:

1. On diet for a reference child in the family –SPAN 2nd Grade. (CHILD DIET)
2. On diet for primary caregiver/parent – SPAN 8th Grade. (CAREGIVER DIET)
3. Measurements of preference, strength of preference, rationale for preference – PREFERENCE (PREFERENCE)
4. Measurements of social needs *at baseline*. AAFP Social Needs Screening Tool (SOCIAL NEEDS)
5. Measurements of demographics, SNAP & WIC status, *at baseline* – DEMOGRAPHICS (DEMOGRAPHICS)
6. Program assessment *at follow-up* (PROGRAM IMPROVEMENT).

Sequence of Measurements

Baseline: Measurements will be taken at baseline, starting -3-4 weeks prior to the start of the program. Many changes have had to be made on timing, but at this point we hope to begin collecting data at the end of March or 1st week of April at the latest. We expect the program to start latest April 26th, but ideally sooner, based on IRB approval dates and other preparation by our community partner.
Baseline measurements: CHILD DIET, CAREGIVER DIET, PREFERENCE, SOCIAL NEEDS, DEMOGRAPHICS.

Post: We will begin post measurements during the 4th week, with a starting date (day 1 of the 4th week onwards) to ensure they are mostly collected by the 7th day of the 4th week.
Post measurements: CHILD DIET, CAREGIVER DIET, PREFERENCE.

Follow-up: We will begin follow-up measurements during the 8th week, with a starting date (day 1 of the 8th week onwards) to ensure they are mostly collected by the 7th day of the 8th week.
Follow-up measurements: CHILD DIET, CAREGIVER DIET, PREFERENCE, PROGRAM IMPROVEMENT.

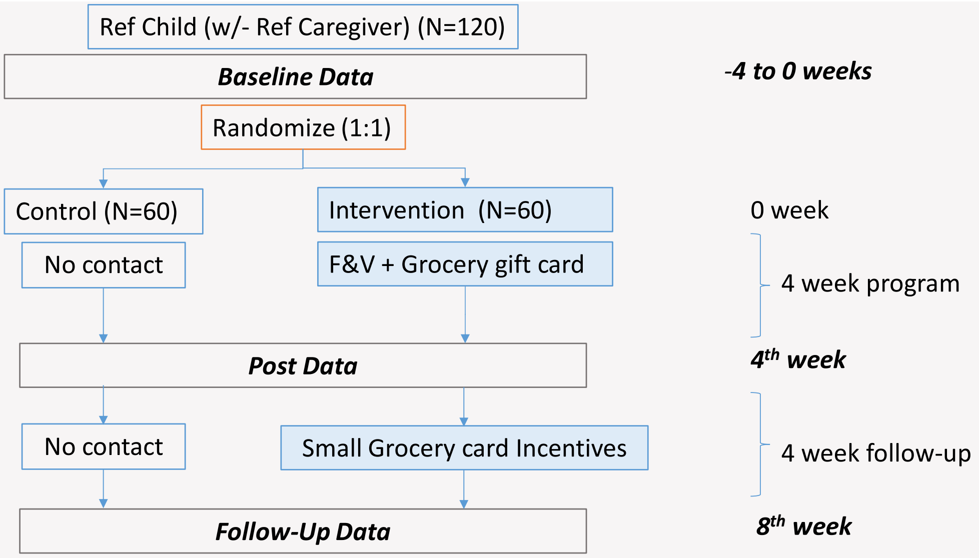

References:

- Thiagarajah K, Fly AD, **Hoelscher DM**, Bai Y, Lo K, Leone A, Shertzer JA. Validating the food behavior questions from the elementary school SPAN questionnaire. J Nutr Ed Behav. *2006*; *38/ 4; S55*.
- Penkilo M, George G, **Hoelscher DM**. Reproducibility of the School Based Nutrition Monitoring Questionnaire among Fourth Grade Students in Texas. J Nutr Ed Behav. *2008*: 40/1; *20-27.*
- **Hoelscher DM, Day RS, Kelder SH** & Ward JL (2003) Reproducibility and validity of the secondary level School-Based Nutrition Monitoring student questionnaire. Journal of American Dietetic Association, 103(2),186-94.

5 Data Analysis

Describe the data analysis plan, including any statistical procedures or power analysis.

To input text, click in the light grey area below.

Given the challenges to recruitment and retention, statistical power was based on a very conservative effective sample size of 120 individuals configuring 4% attrition rate from 125 consented parents. With an allocation ratio of 1 to 1, we expect to retain 60 individuals each in the control and intervention groups.

The primary outcome measure will be SPAN obtained over three assessment waves at baseline, 4-weeks, and 8-weeks in both groups. To accommodate variability in the timing of assessment waves for each participant and any missing data or drop-out, we will utilize mixed linear modeling framework with maximum likelihood estimation. We

will estimate a random intercept and test for a random component of time, i.e. significant individual difference variability in trajectories of healthy eating. The targeted research questions all concern the interaction of grouping indicator and time at specific pairs of assessments. To minimize inflations of type-I error, we will first test the omnibus interaction term. The study will have 80% power to detect an effect size of f = .14 for this omnibus interaction effect, testing differential trajectories for the control and intervention groups, with an alpha = 0.05 and assuming a conservative level of rank-order stability among successive assessment waves, r= .30. This is a small effect size.

25 STUDY ELEMENT IDENTIFICATION

6 Study Elements

Click on the check box (or double click and type an "X" if using Google Docs) each procedure included in your study.

A full description of all study procedures should be provided in the Procedures (Details) section below and/or the applicable supplement form.

☐	**Bio-specimens**	☐	**Biometrics**	☐	**Registry or Repository**
☐	**Focus Group**	☐	**Genetic Analysis**	☐	**Genomic Data Sharing**
☐	**International Research**	☒	**Interview/Survey**	☐	**MRI**
☐	**Protected Health Information**	☒	**Observation**	☐	**Record Review**
☐	**Sensors (Externally Placed)**	☐	**Sensors (Inserted)**	☐	**Video/Audio Recording**
☐	**X-Ray**				

26

7 Study Intervention

Click on the check box (or double click and type an "X" if using Google Docs) if you will implement any of the following interventions.

A full description of all study interventions should be provided in the Procedures (Details) section below and/or the applicable supplement form.

☒ **Behavioral** ☐ **Device** ☐ **Drug/Biologic**

8 Clinical Trial

Click on the following check box (or double click and type an "X" if using Google Docs) if the research meets the below definition of a clinical trial.

☒ **This study meets the definition of a clinical trial according to clinical trials.gov in that it involves one or more human subjects who are prospectively assigned to one or more interventions (which may include placebo or other control) to evaluate the effects of those interventions on health-related biomedical or behavioral outcomes.**

9 Additional Oversight

Click on the check box (or double click and type an "X" if using Google Docs) each activity that requires oversight from additional UT committees.

☐ **Biohazards, Recombinant DNA, or Gene Transfer**

☐ **Energy introduced to the subject (electrical, magnetic, light)**

☐ **Human embryonic, human induced pluripotent, or human totipotent stem cells; or human gametes or embryos**

☐ **Radiation exposure without direct clinical benefit**

10 Alternatives to Participation in This Study

To input text, click in the light grey area below.

There is no alternative to participation.

11 Procedure Description

Describe all study procedures, including a step-by-step outline of what participants will be asked to do or how data will be used. Be sure to describe all of the following in detail, as applicable:

- Provide a description of all research procedures being performed and when they are performed, in sequential order.
- All research measures/tests that will be used and state if questions or measures are standardized or published (upload copies of all surveys, scripts and data collection forms)
- Secondary data or specimens that will be obtained, how they will be collected, and how they will be used
- Where each activity will take place, the duration of each, and who will perform each activity
- Include time commitment of participants

To input text, click in the light grey area below.

Recruitment

1. We will inform potential participants about the program through flyers added to Club on the Go bags beginning a few weeks prior to the start of the program, ideally the last week of March, but depending on final IRB approval. Boys and Girls Club staff will also verbally inform parents about the program when they pick up their Club on the Go Bags.
2. Our partner Boys and Girls Club staff will also create an interest list by calling families to let them know about the research and ask if they wish to learn more. The families will be told that a member of the UT research team will call them to tell them more about the program. If families are interested, they will be added to an interest list that will be provided to the UT research team.
3. As the Boys and Girls Club begins generating an interest list, beginning a few weeks prior to the start of the program, our research team will begin to call those interested

Consent and Assent

1. After identification of those interested, Boys and Girls Club staff will provide the study team with the potential participant's name and phone number. A member of the research team will call the primary caregiver of BGC members, as identified by BGC, to confirm interest and explain program details. They will begin the consenting process by reading the consent form to the primary caregiver. Potential participants will be given time to ask questions about the research and time commitments involved. A verbal consent of the primary caregiver, including date of consent and the name of research team member receiving consent will be captured in the Redcap system which we will be using

for all data collection. Primary caregiver will be verbally provided with the contact information of the research team and the Institutional Review Board for any concerns and questions they may have.

2. After the consent form is read out loud to the primary caregiver, the child will join by speaker phone to provide assent.. The assent form will be read out loud to the child by the research team member. The assent form requires only verbal consent, so the researcher will make sure the child understands what has been read and can ask any questions they may have.

Baseline Measurement

After consent has taken place, a member of the research team will obtain baseline measurements. The call to obtain baseline measurements will take approximately 45 minutes

Randomization

1. Randomization of participants to control or intervention arm will occur after baseline has been taken. Our statistician will generate the sequences which will be input into RedCap by a research manager.
2. Randomization will be implemented with a 1:1 ratio (control: intervention) in blocks of 2 and 4.

Fruits & Vegetable amount provided:

The weekly F&V bag and gift card will be designed to achieve ~1 cup per day F&V per household member (adult or child). We are unable to customize what we are providing based on household size, therefore we will use a 5-person household (e.g. 2 adults and 3 children) as the standard. We will provide 5-10lbs F&V box and gift cards of $10 every week. This should cover 35 cups/70 servings per week. In addition, for every week of weeks 2, 3 and 4 that the participant brings in their progress to goals assessment, we will provide an additional $10 gift card for each of those 3 weeks.

Fruits & Vegetable provider and delivery: The F&V provider is Hardies who will deliver the boxes to BGC Club on the Go sites once a week. Hardies will provide information to study staff 1-2 days prior on the makeup of the F&V box, based on which study staff will print out recipes (examples of the type of recipes provided can be found at: https://www.brighterbites.org/recipe/) which will be included in the box. Study staff will bring the week's recipes and gift cards to the distribution site prior to distribution and provide these to the BGC staff. BGC staff will provide the boxes and gift cards to each person driving up for their Club on the Go bags.

Intervention

1. The intervention arm will receive 4 weeks of premade F&V boxes with recipes

included that are customized to the contents of the bag and a gift card for $10. They will receive the box and gift card at the Club on the Go pickup site every week. They will receive a short assessment form to mark their progress on healthy eating goals in each box (with the recipes). If they complete these and return them when they pick up the bags for weeks 2, 3 and 4, they will receive an additional $10 gift card.

2. Gift cards, though distributed weekly.
3. Everyone in the intervention will also get one cooking incentive valued at between $20-25 each. Cooking incentives will be chosen by participants through the preference survey asked at baseline – (example cooking incentives may include: set of kitchen equipment, cookbook or spice selection).
4. Follow-up period: Everyone in the intervention arm will receive two $5 gift cards sent by mail at week 5 and 6.

Control

1. The control arm will not receive any F&V or gift card during the 4 week intervention period and the subsequent 4 weeks of follow-up.
2. The control arm will receive the same amount in gift cards (total value of $80) mailed to them on the 8th week. Follow-up period: The control arm will not receive any additional incentives during the follow-up period.

33

34

SUBJECT POPULATION

12 Protected Subject Populations

Click on the check box (or double click and type an "X" if using Google Docs) each population, if they are specifically studied for this research.

☐	**Active military personnel**	☒	**Children**	☐	**Decisionally impaired adults**
☐	**Emancipated minors**	☐	**Fetuses**	☐	**Individuals with limited English proficiency**
☐	**Neonates**	☐	**Pregnant Woman**	☐	**Prisoners**
☐	**UT Students**	☐	**UT or Seton Staff/Employees**		

13* Research Participant Information

Describe the research population.

**For multiple research populations (e.g., teachers, students, and parents), copy this section as necessary to describe your population.*

a Participant Group Name

To input text, click in the light grey area below.

Families in the Boys and Girls "Club on the Go" program

b Minimum Age

To input text, click in the light grey area below.

Kindergarten for child participants; 18 for adult participants

c Maximum Age

To input text, click in the light grey area below.

Fifth grade for child participants; no maximum for adult participants

d Inclusion Criteria

To input text, click in the light grey area below.

1. Families must be participants in the Boys and Girls "Club on the Go" program. They could sign up to become a participant while we are recruiting.
2. Caregiver must be able to come to the same "Club on the Go" site every week.
3. At least one child in the family must be k-5 student.
4. Caregiver must be able to read and write in English or Spanish to answer assessments.

e Exclusion Criteria

To input text, click in the light grey area below.

1. Reference child with any medical condition in which they have to follow a specific diet.
2. Caregiver cannot read and write in English or Spanish.
3. Reference child not living with caregiver for majority of time (75% or greater).
4. Families that do not meet the inclusion criteria

f Additional Population Information

To input text, click in the light grey area below.

37

14 Total Sample Size

To input text, click in the light grey area below.

Our goal is 150 participants recruited which is ambitious given the numbers of potential participants currently driving up to Boys and Girls Club, Club on the Go sites. It is reasonable to expect we will be able to include 125 consented to the study. The power analysis assumes 4% attrition, and uses N of 120.

38

15 Sample size rationale

To input text, click in the light grey area below.

Given the challenges to recruitment and retention, statistical power was based on a very conservative effective sample size of 120 individuals configuring 4% attrition rate from 125 consented parents.

The primary outcome measure will be SPAN obtained over three assessment waves at baseline, 4-weeks, and 8-weeks in both groups. To accommodate variability in the timing of assessment waves for each participant and any missing data or drop-out, we will utilize mixed linear modeling framework with maximum likelihood estimation. We will estimate a random intercept and test for a random component of time, i.e. significant individual difference variability in trajectories of healthy eating. The targeted research questions all concern the interaction of grouping indicator and time at specific pairs of assessments. To minimize inflations of type-I error, we will first test the omnibus interaction term. The study will have 80% power to detect an effect size of f = .14 for this omnibus interaction effect, testing differential trajectories for the control and intervention groups, with an alpha = 0.05 and assuming a conservative level of rank-order stability among successive assessment waves, r= .30. This is a small effect size.

39

SCREENING AND RECRUITMENT

16 Identification and Screening

Click on the check box (or double click and type an "X" if using Google Docs) if true.

☒ **This study involves obtaining information or biospecimens for the purpose of screening, recruiting or determining eligibility of prospective subjects prior to informed consent by either:**

1. Oral or written communication with the prospective subject or LAR
2. By accessing records containing identifiable private information or stored identifiable biospecimens.

40

17 Identification and/or Screening Procedures

Describe the identification and/or screening procedures below.
To input text, click in the light grey area below.

A few weeks prior to the start of the program, our partner Boys and Girls Club staff will create an interest list by calling and asking families if they wish to learn more. The families will be told that a member of the UT research team will call them to tell them more about the program. If families are interested, they will be added to an interest list that will be provided to the UT research team.

41

18 Recruitment Overview

Click on the check box (or double click and type an "X" if using Google Docs) all recruitment methods utilized for this research.

☐	E-mail	☒	Flyer
☒	In-Person	☐	Letter
☐	Social Media	☐	Research Pool
☒	Telephone/Text	☐	Snowball Sampling
☐	Web-post	☒	Word of Mouth

42

19 Describe the recruitment process, including where recruitment will take place.

Describe the recruitment procedures below.
To input text, click in the light grey area below.

Recruitment

The Boys and Girls Club has a "Club on the Go" program already implemented at elementary school sites and they plan on continuing this program through the period of this study. We are partnering with them for the entire study, and they will help in recruitment and distribution components the program. All recruitment questions and adding to interest list occur as over the phone or as cars drive up for a Club on the Go bag.

1. We will inform potential participants about the program through flyers added to

Club on the Go bags a few weeks prior to the start of the program. Boys and Girls Club staff will also verbally inform parents about the program during regular check-in phone calls or when they pick up their Club on the Go Bags.

2. Beginning a few weeks prior to the start of the program, our partner Boys and Girls Club staff will create an interest list by calling or asking families at pick-up if they wish to learn more about a research program. The families will be told that a member of the UT research team will call them to tell them more about the program. If families are interested, they will be added to an interest list that will be provided to the UT research team.
3. As the Boys and Girls Club begins generating an interest list, our research team will begin to call those interested.

43

44

OBTAINING INFORMED CONSENT

20 Consent Overview

Click on the check box (or double click and type an "X" if using Google Docs) all applicable items.

- ☐ **Obtaining Written Informed Consent**
- ☒ **Requesting a Waiver of Documentation of Informed Consent**
- ☐ **Requesting a Waiver of Informed Consent**
- ☐ **Requesting an Alteration of the Required Elements of Informed Consent**
- ☒ **Obtaining Child Assent**
- ☐ **Obtain Consent Using a Short Form with a Witness**

45

21 Consent and Assent Processes

Provide a detailed description of the consent process including who will obtain consent, where, and when consent will occur in such a manner that participants have sufficient time for adequate consideration.

To input text, click in the light grey area below.

Due to the low-risk nature of this study, our intention is to obtain consent and assent via telephonic means. To obtain consent, a member of the research team will call interested primary caregivers of BGC members, as identified by BGC, and confirm interest in the study. The consent form contents will be read to that possible participant. Possible participants will be given the opportunity to ask any questions about the study should they have them, and offered time for consideration prior to

obtaining verbal consent. Verbal consent will be obtained via telephonic engagement and documented by the researcher in Redcap, a system which will be used for data collection.
Once the primary caretaker agrees, verbal assent will be obtained from the child. The child will be on speaker phone during the same call to provide verbal assent. The assent form contents will be read to the child. The child will be given the opportunity to ask questions about the study should they have them. The assent form requires only verbal consent which will be documented by the researcher in Redcap. There is no option to receive a written copy of the consent.

46

22 Consent and Translation

Click on the check box (or double click and type an "X" if using Google Docs) to indicate that consent will be translated.

☒ **The study population will likely include participants whose limited English speaking status requires translation of the consent form.**

Translation Process

Click on the check box (or double click and type an "X" if using Google Docs) that best describes the translation process, either 21 or 22.

23 ☐ **The consent documents will be translated by a certified translator.**

24 ☒ **A non-certified translator will translate the consent documents.**

If selected, complete the next two questions below.

i **Describe the translator's qualifications**

To input text, click in the light grey area below.

Two English fluent and native Spanish speakers within research personnel

ii ☒ **Another individual will confirm that the translation is accurate and appropriate.**

47

Waiver of Documentation of Informed Consent

To approve a waiver of documentation of informed consent, one of the following options below must be justified by the researcher.

Only complete the sections below if requesting a waiver of documentation of informed consent. *If not requesting a waiver of documentation of consent, skip to 27.*

Please choose one waiver option and provide additional information as prompted. The Office of Research Support and Compliance recommends using Waiver Option 2 in most cases.

25 **Waiver Option 1**

Provide confirmation for the following criteria and follow the additional instructions.

Additional Instructions:
1. Include this choice in the informed consent form.
2. Articulate the destruction process for signed consent forms in the privacy and confidentiality section.

Click on the check box (or double click and type an "X" if using Google Docs).

a ☐ **The only record linking the subject and the research would be the consent document.**

b ☐ **The principal risk would be potential harm resulting from a breach of confidentiality.**

c ☐ **Each subject will be asked whether the subject wants documentation linking the subject with the research, and the subject's wishes will govern.**

26 **Waiver Option 2**

Provide confirmation for the following criteria and follow the additional instructions.

Click on the check box (or double click and type an "X" if using Google Docs).

a ☒ **The study is minimal risk.**

b ☒ **Written consent would not be required outside the research context.**

27 **Waiver Option 3**

Provide confirmation for the following criteria and provide additional information as requested.

Click on the check box (or double click and type an "X" if using Google Docs).

a ☐ **The subjects or legally authorized representatives are members of a distinct cultural group or community in which signing forms is not the norm**

b **Describe the cultural group or community.**
To input text, click in the light grey area below

c ☐ **The research presents no more than minimal risk of harm to subjects.**

d ☐ **There is an appropriate alternative mechanism for documenting that informed consent was obtained.**

e **Describe mechanism for documenting that informed consent was obtained**

To input text, click in the light grey area below

48

Waiver or Alteration of Informed Consent

To approve a waiver or alteration of informed consent all of the following criteria below must be justified by the researcher.

Only complete the sections below if requesting a waiver of informed consent. *If not requesting a waiver or alteration of consent, skip to 31.*

28 **The research involves no more than minimal risk to the subjects.**

To input text, click in the light grey area below

29 **The waiver or alteration will not adversely affect the rights and welfare of the subjects.**

To input text, click in the light grey area below

30 **The research could not practicably be carried out without the waiver or alteration (it is impracticable to perform the research if obtaining informed consent is required and not just impracticable to obtain consent).**

To input text, click in the light grey area below

31 **If the research involves using identifiable private information or identifiable biospecimens, the research could not practicably be carried out without using such information or biospecimens in an identifiable format.**

To input text, click in the light grey area below.

49

50

Deception and Debriefing

Only complete the sections below if requesting an alteration of informed consent that involves deceiving research participants. *If this study does not involve deception, skip to 35.*

See IRB Policies and Procedures Section 15 for a description of deception.

Click on the check box (or double click and type an "X" if using Google Docs).

32 ☐ **It is appropriate to provide additional pertinent information to the subject after research activities are complete (e.g., the researcher needed to deceive to subject to the nature of the study).**

33 ☐ **Research participants will have the opportunity to withdrawal their data during the debriefing.**

34 **Describe the nature of deception and why it is necessary to conduct the research.**

To input text, click in the light grey area below.

35 **Describe debriefing procedures.**

To input text, click in the light grey area below.

51 BENEFITS

36 **Benefits to Society**

Describe the scientific and societal benefit(s) below.

To input text, click in the light grey area below.

By demonstrating improvement of diet by providing low-resourced families not only the opportunity to try more fruits and vegetables to improve their family's diet.

52

Benefits to Participants

Click on the applicable check box (or double click and type an "X" if using Google Docs).

37 ☐ **There is no anticipated direct benefit to participants.**

38 ☒ **There are anticipated benefits to participants.**

39 **If applicable, describe the potential direct benefits to participants.**

To input text, click in the light grey area below.

Participants will receive free produce and gift card while participating in this study.

RISKS

40 **Describe the risks associated with each activity in this research**

To input text, click in the light grey area below.

This is a minimum risk study. The primary risk is the potential loss of confidentially. Additionally, as we do not have control over the specific contents of the F&V bag, we are not able to accommodate individual conditions such as food allergies.

41 **Describe how each risk is mitigated/minimized.**

To input text, click in the light grey area below.

Risk will be minimized by using a secure REDCap database for all data collection and storage. To reduce the risk of possible exposure to food allergens participating caregivers will be alerted to this fact that we cannot accommodate specific dietary needs regarding the F&V bag and may choose not to participate

Data Safety Monitoring

For additional information regarding data safety monitoring boards and data safety monitoring plans, please see Section 21 of our Policies and Procedures.

Click on the check box (or double click and type an "X" if using Google Docs).

42 ☒ **This study is minimal risk and does not require a Data Safety Monitoring Plan (DSMP) or a Data Safety Monitoring Board (DMSB).**

43 ☐ **This study does not have a Data Safety Monitoring Board, but researchers have an internal plan/policy to monitor for safety.**

Complete Data Safety Monitoring Details (44-51).

44 ☐ **This study has a Data Safety Monitoring Board (DSMB).**

Complete Data Safety Monitoring Details (44-51) or upload this study's Data Safety Monitoring Board's charter.

Data Safety Monitoring (Details)

45 **How is safety information collected?**

To input text, click in the light grey area below.

46 **When will safety data collection start (for each participant or for the whole study, as applicable)?**

To input text, click in the light grey area below.

47 **How frequently will safety data be collected?**

To input text, click in the light grey area below.

48 **Who will review the data for safety?**

To input text, click in the light grey area below.

49 **How frequently will data be monitored for safety concerns?**

To input text, click in the light grey area below.

50 **What data will be reviewed?**

To input text, click in the light grey area below.

51 **State the frequency or periodicity of the review of cumulative data?**

To input text, click in the light grey area below.

52 **State any conditions that would trigger an immediate suspension of the research.**

To input text, click in the light grey area below.

58

Early Withdrawal

Only complete this section if there are planned conditions under which a participant will be withdrawn from the study. *If not applicable, skip to 56.*

Include this information in your consent form.

53 **List the criteria for withdrawing individual participants from the study (e.g., safety or toxicity concerns, emotional distress, inability to comply with the protocol, or requirements from study sponsor).**

To input text, click in the light grey area below.

54 **Describe any necessary procedures for ensuring the safety of a participant who has withdrawn early.**

To input text, click in the light grey area below.

55 **Describe any pre-specified criteria for stopping or changing the study protocol due to safety concerns.**

To input text, click in the light grey area below.

59 REQUIRED DISCLOSURES

Required Consent Disclosures

Identify each element below that may require additional information to be disclosed in the consent form.

Click on the check box (or double click and type an "X" if using Google Docs).

56 ☒ **It is reasonable that researchers could discover or suspect child or elder abuse.**

57 ☐ **It is reasonable that researchers could learn of an incident that could require reporting under Title IX.**

58 ☐ **It is reasonable that researchers could discover incidental findings or other information of medical interest about a participant's previously unknown condition.**

59 **Articulate methods for addressing and reporting incidental findings, if applicable.**

To input text, click in the light grey area below.

If a member of the research team has reason to suspect child abuse, they will report concerns to the PI who will work with the team member to determine the appropriate course of action, including possible reporting to DFPS.

60

61

62

PRIVACY AND CONFIDENTIALITY

60 **Privacy**

Describe how you will protect the identity and privacy of study participants during each phase of research. Privacy focuses on the individual participants rather than data. In this section, researchers should focus on issues such as where research activities take place and how participant involvement is protected from non-participants.

Describe methods to ensure participants' privacy during identification, recruitment, screening, the consent process, the conduct of the study, and dissemination of data.

To input text, click in the light grey area below.

The Boys and Girls Club (BGC) will create an interest list by calling or asking families in person if they wish to know more during their pick-up of Club on the Go bags. Families will be informed that they will be contacted by a member of the UT research team. Since these screening activities will take place over the phone or during regular drive-through pick-up services, potential participants will be in their car or home. UT research personnel will reach out to the primary caregiver of the family to confirm interest, explain further details, and obtain verbal consent. This will be done over the phone using the phone number provided by the BCG's interest list. Both locations are reasonably considered to be in the in the interest of maintain the privacy of participants.

63

64

Confidentiality and Data Security Plan

Click on the check box (or double click and type an "X" if using Google Docs) that best describes the confidentiality and data security plan and provide additional details regarding how you will protect the confidentiality of data or address confidentiality concerns.

61 ☐ **Identifiers will be coded to protect confidentiality.**

61a If true, state how data is coded and where identifiers are stored.

To input text, click in the light grey area below.

62 ☒ **Identifiable data will be destroyed.**

62a If true, describe destruction plan and timeline

To input text, click in the light grey area below.

All identifiers will be removed from the data at the end of the study.

63 ☐ **Identifiable data will not be destroyed.**

63a If true, provide rationale for retaining identifiable data indefinitely.

To input text, click in the light grey area below.

64 Data Access

Click on the check box (or double click and type an "X" if using Google Docs) for each group of individuals that will have access to study data.

If you plan on creating a repository, complete the repository form as well.

☒ **Study Team Members**

☐ **External Collaborators**

☐ **Data coordinating center**

☐ **Sponsor**

☐ **Future Sharing with other researchers**

☒ **Others**

Describe below. To input text, click in the light grey area below.

We will share data required to distribute specific intervention elements with B&G club.

65 Describe data sharing plan for each group checked above and state whether researchers plan on sharing identifiable, coded, or de-identified data

To input text, click in the light grey area below.

All data will be captured in REDCap. Only members of the UT research team will have direct access to the REDCap database. Distribution list information will be shared with B&G club manager through UT Box.

67

Certificate of Confidentiality

Click on the check box (or double click and type an "X" if using Google Docs) to identify each element below that may require additional information to be disclosed in the consent form.

If a Certificate of Confidentiality is not applicable for this study, skip to 68.

66 ☐ **The study requires a Certificate of Confidentiality.**

67 ☐ **NIH has issued a Certificate of Confidentiality for this study.**

68 ☐ **A Certificate of Confidentiality has not been obtained, but there are plans to apply for one.**

68 # COMPENSATION AND COSTS

Compensation

Click on the check box (or double click and type an "X" if using Google Docs).

69 ☒ **Subjects receive compensation.**

70 ☐ **Subject will not receive compensation.**

Skip to question 74 if subjects will not receive compensation.

71 **Total Amount of Compensation**

To input text, click in the light grey area below.

All participants whether in intervention or control will be provided a possible total of $45.00 in gift cards ($15.00 for completion of each study call (3 total) and associated measures) to compensate for what we assess will be 2.5 hours of time to complete study measures. Gift cards will be distributed at study end based on the total number of surveys completed.

72 **Type of Compensation**

Click on the check box (or double click and type an "X" if using Google Docs) for each form of compensation that will be provided.

☐ Cash	☐ Check	☒ Gift Card
☐ Course Credit	☐ ClinCard	☒ Tango Card
☒ Other		

Describe, To input text, click in the light grey area below.

1. Each participant, whether in control or intervention will receive up to $45.00 in generic gift cards to compensate for time spent in providing

input on surveys. (see above).
2. The intervention includes providing produce and gift cards worth a maximum of $70.00 for individuals.
3. Individuals in the intervention will also receive a “cooking incentive” (for example a spice kit) valued between $20-25. Those randomized to control will not have the option to receive this incentive.
4. Individuals in the control group will receive $80 worth of gift carts at the end of the study to roughly match the value of the F&V/gift cards received by the intervention group.

69

73 **Proration Schedule**

To input text, click in the light grey area below.

70

74 ☒ **Amount of compensation and its form is reasonable for this population for the activities requested of them.**

71

75 **Costs**

Click on the check box (or double click and type an “X” if using Google Docs) each applicable item regarding costs.

- ☒ **Participants will have no costs associated with this study**
- ☐ **Standard of care procedures contributing to study data**
- ☐ **Research procedures not associated with standard of care**
- ☐ **Administration of drugs / devices**
- ☐ **Study drugs or devices**
- ☐ **Transportation and parking**

76 **Describe all costs below.**

To input text, click in the light grey area below.

72
