# Supplementary material for: Effect of Brief Produce Exposure and Unconstrained Grocery Gift Cards on Caregiver Influence on Diet of Elementary Age Children: A Randomized Clinical Trial
Source: JAMA Netw Open. 2022 May 27;5(5):e2212973. doi: 10.1001/jamanetworkopen.2022.12973 (PMC9142863; doi:10.1001/jamanetworkopen.2022.12973)
Supplement: Supplement 2. — Data Sharing Statement [file jamanetwopen-e2212973-s002.pdf]

Data Sharing Statement

Kahlon M, Aksan NS, Aubrey R, et al. Effect of brief produce exposure and unconstrained grocery gift cards on caregiver influence on diet of elementary age children: a randomized clinical trial. *JAMA Netw Open*. 2022;5(5):e2212973. doi:10.1001/jamanetworkopen.2022.12973

Data
Data available: Yes
Data types: Deidentified participant data, Data dictionary **How to access data:** Laura.gougeon@austin.utexas.edu **When available:** beginning date: 09-01-2022

Supporting Documents
Document types: Informed consent form
How to access documents: mkahlon@austin.utexas.edu
When available: With publication

Additional Information
Who can access the data: Laura.gougeon@austin.utexas.edu **Types of analyses:** For exploratory analyses
Mechanisms of data availability: Signed data agreement
